# Genome-Wide Association Study Reveals Four Loci for Lipid Ratios in the Korean Population and the Constitutional Subgroup

**DOI:** 10.1371/journal.pone.0168137

**Published:** 2017-01-03

**Authors:** Taehyeung Kim, Ah Yeon Park, Younghwa Baek, Seongwon Cha

**Affiliations:** Mibyeong Research Center, Korea Institute of Oriental Medicine, Yuseong-gu, Daejeon, Republic of Korea; Loyola University Chicago, UNITED STATES

## Abstract

Circulating lipid ratios are considered predictors of cardiovascular risks and metabolic syndrome, which cause coronary heart diseases. One constitutional type of Korean medicine prone to weight accumulation, the Tae-Eum type, predisposes the consumers to metabolic syndrome, hypertension, diabetes mellitus, etc. Here, we aimed to identify genetic variants for lipid ratios using a genome-wide association study (GWAS) and followed replication analysis in Koreans and constitutional subgroups. GWASs in 5,292 individuals of the Korean Genome and Epidemiology Study and replication analyses in 2,567 subjects of the Korea medicine Data Center were performed to identify genetic variants associated with triglyceride (TG) to HDL cholesterol (HDLC), LDL cholesterol (LDLC) to HDLC, and non-HDLC to HDLC ratios. For subgroup analysis, a computer-based constitution analysis tool was used to categorize the constitutional types of the subjects. In the discovery stage, seven variants in four loci, three variants in three loci, and two variants in one locus were associated with the ratios of log-transformed TG:HDLC (log[TG]:HDLC), LDLC:HDLC, and non-HDLC:HDLC, respectively. The associations of the GWAS variants with lipid ratios were replicated in the validation stage: for the log[TG]:HDLC ratio, rs6589566 near *APOA5* and rs4244457 and rs6586891 near *LPL*; for the LDLC:HDLC ratio, rs4420638 near *APOC1* and rs17445774 near *C2orf47*; and for the non-HDLC:HDLC ratio, rs6589566 near *APOA5*. Five of these six variants are known to be associated with TG, LDLC, and/or HDLC, but rs17445774 was newly identified to be involved in lipid level changes in this study. Constitutional subgroup analysis revealed effects of variants associated with log[TG]:HDLC and non-HDLC:HDLC ratios in both the Tae-Eum and non-Tae-Eum types, whereas the effect of the LDLC:HDLC ratio-associated variants remained only in the Tae-Eum type. In conclusion, we identified three log[TG]:HDLC ratio-associated variants, two LDLC:HDLC ratio-associated variants, and one non-HDLC:HDLC-associated variant in Koreans and the constitutional subgroups.

## Introduction

Blood cholesterol and lipids are heritable risk factors of coronary artery disease (CAD), which causes morbidity and mortality among adults [1,2]. In a meta-analysis including 188,577 individuals from genome-wide association studies (GWASs) and Metabochip arrays, 157 loci were found to be associated with lipid levels including levels of triglycerides (TG), total cholesterol, HDL cholesterol (HDLC), and LDL cholesterol (LDLC). Specific loci among them were also found to be associated with CAD, type 2 diabetes, blood pressure, waist-to-hip ratio, and body mass index [3]. Many large-scale association studies have focused only on the associations between variants and traditional lipid levels.

However, lipid cholesterol ratios (log[TG]:HDLC and LDLC:HDLC) have been suggested to be better predictors of the development of coronary heart disease (CHD) than traditional lipid levels in the Framingham Offspring Study [4]. A high log[TG]:HDLC ratio strongly correlates with smaller, denser LDL particles and is significantly associated with increased extent of coronary disease in the coronary angiogram [5,6]. The LDLC:HDLC ratio can predict CHD development with higher hazard ratio than LDLC and HDLC levels and has been found to be an independent predictor for acute myocardial infarction in the Japanese population [4,7]. The non-HDLC:HDLC ratio is also suggested to be a better predictor of CHD risk than LDLC levels in a follow-up study for a mean of 4.8 years in the Swedish National Diabetes Register and has been found to be a stronger marker of metabolic syndrome and insulin resistance in Korean adults than apolipoprotein B/apolipoprotein A1 [8,9].

The Sasang constitutional medicine divides human beings into four types (Tae-Eum (TE), So-Eum, So-Yang, and Tae-Yang) according to their sensitivity to certain groups of herbs and medicines and the equilibrium between their internal organic functions, physical features, and psychological characteristics [10]. The TE type predisposed to increased abdominal obesity has a higher risk of metabolic syndrome, diabetes mellitus, hypertension, and insulin resistance than the other types [11–15]. In terms of genetic predisposition to hypo-HDLC-emia and hyper-triglyceridemia, individuals of the TE type are more likely to have low serum HDLC levels associated with Apolipoprotein A-V (*APOA5*) ‒1131T>C polymorphism [16]. In our previous study, we reported that the minor allele effect of three variants (near *APOA5*, apolipoprotein C1 (*APOC1*), and lipase C) made the TE type more susceptible to increased TG and LDLC levels and decreased HDLC levels; on the other hand, in the non-TE type, the adverse effects are compensated for by protective effects, leading to a neutral influence on the lipid risk [17].

Many loci associated with lipid levels have been identified using GWAS, and differential effect sizes of lipid-associated loci in constitutional subgroups have been suggested in previous reports. However, the loci associated with lipid ratios have not been fully elucidated. Therefore, in this study, to identify new loci associated with lipid ratios, we performed GWASs and confirmed the association of the candidate loci in an independent population. Because of the genetic discrepancy of lipid traits, subgroup analysis was additionally performed for the TE type and non-TE type subjects. Our study indicates that GWAS for lipid ratios could be a good approach to find novel loci that have not yet been discovered by GWAS for individual lipid traits.

## Materials and Methods

### Study subjects

We selected 5,292 Korean subjects (2,621 men and 2,671 women) who were a part of community-based cohort studies from two regions in South Korea (Ansan and Ansung) from 2009 to 2012 for the Korean Genome and Epidemiology Study (KoGES) [18] for the GWAS. For the independent replication analysis, 2,567 Korean subjects (910 men and 1,657 women) were recruited from 22 oriental medical clinics for the Korea medicine Data Center (KDC) from 2006 to 2012. None of the subjects from the KoGES or KDC populations had a history of cancer treatment, postmenopausal hormonal therapy, and professional diagnosis or medication for dyslipidemia. Additionally, the KoGES subjects did not include those with low-quality genome-wide genotype data caused by gender inconsistencies, cryptic relatedness, and problems with genotype call rate and sample contamination as previously described [18]. All the subjects provided written informed consent to participate in the study, and the study was approved by the Institutional Review Board of the Korea Institute of Oriental Medicine.

The subjects (n = 5,229 in KoGES and n = 2,088 in KDC) were analyzed using an integrated diagnostic model consisting of face, body shape, voice, and questionnaire information, i.e., the Sasang Constitutional Analysis Tool (SCAT), in order to provide a basis for discriminating the constitutional types based on the probability values for each Sasang constitutional type [19]. The 63 KoGES and 479 KDC subjects were excluded after the SCAT analysis, due to missing data in the four components of the SCAT or low-quality data for facial pictures and vocal records [19]. Based on the tertiles of the SCAT probability values for the TE type, we divided the study subjects into 3 subgroups. The subjects on the top tertile were designated as the TE type (TE: n = 1,743 in the KoGES; n = 696 in the KDC), and those on the bottom tertile were designated as the NTE type (NTE: n = 1,743 in the KoGES; n = 696 in the KDC). To increase the reliability for the SCAT-determined constitutional type, the subjects with the middle tertile values were not used in the sub-group analysis based on TE type.

### Genotyping

Genome-wide single nucleotide polymorphism (SNP) genotyping of the 5,292 KoGES subjects was performed using the Affymetrix Human SNP array 5.0 (Affymetrix, Santa Clara, CA) as previously described [18]. Of the 500,568 SNPs examined, those exhibiting high missing call rates (>5%), low minor allele frequencies (<0.05), or significant deviations from the Hardy-Weinberg equilibrium (HWE; p < 0.0001) were excluded for quality control, and the remaining 310,746 SNPs were subjected to further analyses.

The genotypes of ten variants that passed a statistical cut-off p-value for association with lipid ratios (rs180349, rs6589566, rs4244457, rs6586891, rs8067076, rs6501843, and rs2885819 for log[TG]:HDLC ratio, rs4420638, rs17445774, and rs2304072 for LDLC:HDLC ratio, and rs180349 and rs6589566 for non-HDLC:HDLC ratio) in the initial GWAS were determined in the 2,567 KDC subjects. For 805 subjects, the genotypes were determined by extracting the genotypes of 10 SNPs from Affymetrix SNP array, and for 1,762 subjects, they were determined by performing TaqMan® assay on three SNPs (rs180349, rs4244457, and rs17445774) in the Fluidigm BioMark^TM^ System (Fluidigm, South San Francisco, CA) or melting analysis of an unlabeled oligonucleotide probe (UOP) applied during PCR on the remaining seven SNPs [20]. The detailed process of genotyping using a UOP for the variant has been described in a previous report [21]. Nine variants except rs180349 were within the HWE in the KDC population (p > 0.01). Therefore, we performed association analyses using the nine SNPs in the KDC and combined populations.

### Statistical analyses

During the discovery stage, GWAS was performed for identifying the variants associated with lipid ratios (log[TG]:HDLC ratio, LDLC:HDLC ratio, and non-HDLC:HDLC ratio) by linear regression analysis in an additive model using PLINK version 1.07 (http://pngu.mgh.harvard.edu/purcell/plink/) [22], with adjustment for age, sex, and recruitment region. Quantile–quantile plots for each lipid ratio were constructed with the distribution of the observed p-values against the theoretical distribution of the expected p-values. The genomic control inflation factors (λ) for the GWAS of each lipid ratio were checked for potential p-value inflation. Manhattan plots for the lipid ratios were generated using R version 3.0.2 software (http://www.r-project.org/), and regional plots with a 1-megabase (Mb) window centered at the variant with the peak SNP were constructed using the web-based LocusZoom tool [23].

In the replication analysis, linear regression analyses of the lipid ratios were performed to confirm the association of the GWAS SNPs in the KDC population, with adjustment for age and sex using R version 3.0.2. Chi-squared test was used to determine whether the GWAS SNPs deviated from HWE in the KDC population. Linkage disequilibrium (LD; Lewontin’s D’ = D/|Dmax| and r^2^) was determined using Haploview version 4.2 (Daly Lab at the Broad Institute, Cambridge, MA) [24]. The interaction between TE category and lipid ratio-associated variants was assessed by adding an interaction term in the linear regression model. In the subgroup analysis according to TE and NTE types, the associations of the lipid ratios shown in all the subjects were revaluated in two populations, with adjustment for age and sex.

The association results from the GWAS and replication analysis were combined using Comprehensive Meta-Analysis program version 2.0 (Biostat, Englewood, NJ) in a random effect model by the DerSimonian and Laird method [25].

Genome-wide significance at the Bonferroni-corrected level (0.05/310,746 SNPs) and nominal significance (cut-off) in the GWAS (stage 1) were defined at p < 1.6 × 10^−7^and p < 5.0 × 10^−6^, respectively, and we regarded a p-value of 0.05 as the cut-off in the replication (stage 2) and the constitutional subgroup analyses. The SNPs in the combined analysis of GWAS and replication analysis were considered significant when the p-values showed traditional genome-wide significance, i.e. p < 5.0 × 10^−8^. The SNPs in the combined analysis of the constitutional subgroup were considered significant when p-values were at the Bonferroni-corrected level (0.05/5 SNPs), i.e. p < 1.0 × 10^−2^.

## Results

### Characteristics of the study subjects

We analyzed the effects of the common variants on lipid ratios such as the log[TG]:HDLC ratio, LDLC:HDLC ratio, and non-HDLC:HDLC ratio in two independent Korean populations as follows: GWAS in the KoGES population comprising 5,292 individuals (discovery stage: stage 1) and replication analysis in the KDC population comprising 2,567 individuals (replication stage: stage 2). The characteristics of the two populations, including traits related to dyslipidemic risk, are presented in Table 1. The KoGES population included older individuals and a higher proportion of men than the KDC population. Subjects with the TE type tended to have higher values of BMI and waist circumference as well as dyslipidemic traits including lipid ratios than those with NTE type, which are consistent with the results of previous reports [26,27].

**Table 1:** Characteristics of the study subjects

Characteristic	KoGES				KDC			
	All	TE^a^	NTE^a^	*P*-value	All	TE^a^	NTE^a^	*P*-value
n	5,292	1,743	1,743	‒	2,567	696	696	‒
Male (%)	48.49	53.35	44.23	7.14 × 10^−8^	35.45	44.25	25.29	1.09 × 10^−13^
Age (y)	60.44 ± 8.59	61.24 ± 8.65	59.51 ± 8.54	6.69 × 10^−10^	47.21 ± 15.85	52.01 ± 16.56	42.62 ± 14.45	<2.2 × 10^−16^
BMI (kg/m^2^)	24.42 ± 3.10	27.08 ± 2.58	21.89 ± 2.05	<2.2 × 10^−16^	23.23 ± 3.270	26.07 ± 2.73	20.57 ± 2.30	<2.2 × 10^−16^
WC (cm)	86.40 ± 8.48	93.30 ± 6.73	79.64 ± 6.30	<2.2 × 10^−16^	83.43 ± 9.799	91.91± 7.75	75.43 ± 6.82	<2.2 × 10^−16^
TG (mg/dL)	142.8 ± 98.17	156.4 ± 103.6	133.4 ± 103.9	<2.2 × 10^−16^	120.3 ± 76.93	146.1 ± 87.30	91.85 ± 53.01	<2.2 × 10^−16^
LDLC (mg/dL)	120.8 ± 32.52	121.0 ± 32.73	120.9 ± 33.06	0.806	107.0 ± 29.33	112.7 ± 29.54	100.5 ± 28.09	2.50 × 10^−15^
HDLC (mg/dL)	45.82 ± 12.25	43.83 ± 10.84	48.07 ± 13.36	<2.2 × 10^−16^	47.60 ± 12.35	43.74 ± 10.58	52.57 ± 12.55	<2.2 × 10^−16^
non-HDLC (mg/dL)	148.0 ± 33.63	149.5± 33.40	146.5 ± 33.98	1.08 × 10^−2^	137.3 ± 32.96	146.0 ± 33.04	126.1 ± 30.52	<2.2 × 10^−16^
TG:HDLC ratio	3.513 ± 3.20	3.942 ± 3.20	3.196 ± 3.64	<2.2 × 10^−16^	2.892 ± 2.422	3.693 ± 2.69	1.961 ± 1.58	<2.2 × 10^−16^
LDLC:HDLC ratio	2.793 ± 0.99	2.895 ± 0.96	2.691 ± 1.04	1.11 × 10^−11^	2.398 ± 0.9025	2.699 ± 0.88	2.023 ± 0.76	<2.2 × 10^−16^
non-HDLC:HDLC ratio	3.452 ± 1.175	3.611 ± 1.16	3.290 ± 1.20	<2.2 × 10^−16^	3.107 ± 1.174	3.528 ± 1.15	2.554 ± 0.94	<2.2 × 10^−16^

Values are presented as mean ± standard deviation.

^a^After removing individuals with missing or low-quality data of the SCAT values, the all subjects (n = 5,229 in KoGES and 2,088 in KDC) were used in the TE and NTE typing, based on the tertiles of the TE probability values via the SCAT.

P-values for comparing TE and NTE types were estimated using the Wilcoxon rank sum test except for male (%), for which the chi-squared test was used.

Abbreviations: KoGES, Korean Genome and Epidemiology Study; KDC, Korea medicine Data Center; BMI, body mass index; WC, waist circumference; TG, triglyceride; LDLC, LDL cholesterol; HDLC, HDL cholesterol; non-HDLC, non-HDL cholesterol.

### Common variants associated with lipid ratios in all the subjects

We performed GWAS to identify the genetic variants associated with lipid ratios in the KoGES population in stage 1. The quantile–quantile plots presented deviations only in the extreme tail probabilities between the distributions of the expected and observed p-values (λ = 1.019 for log[TG]:HDLC ratio, λ = 0.990 for LDLC:HDLC ratio, and λ = 1.002 for non-HDLC:HDLC ratio), indicating that population stratification effects can be considered negligible (S1 Fig). Significant genome-wide association signals were found in chromosome 11 (rs180349 and rs6589566) for the log[TG]:HDLC and non-HDLC:HDLC ratios and in chromosome 19 (rs4420638) for the LDLC:HDLC ratio; in addition, five SNPs for log[TG]:HDLC and two SNPs for LDLC:HDLC *p* < 5.0 × 10‒^6^ in stage 1 (S2 Fig and Table 2).

**Table 2:** Linear regression analysis for the lipid ratio

SNP	Chr	Gene^a^	Allele	MAF	Stage 1			Stage 2			Combined analysis	
					n	beta ± se	*P*-value	n	beta ± se	*P*-value	beta ± se	*P*-value
**log[TG]:HDLC ratio**												
rs6589566	11	*APOA5*	A>G	0.215	5283	0.002215 ± 0.0003512	3.08 × 10^−10^	2558	0.001820 ± 0.0004809	1.58 × 10^−4^	0.002078 ± 0.0002836	2.39 × 10^−13^
rs4244457	8	*LPL*	C>T	0.328	5276	-0.001574 ± 0.0003073	3.12 × 10^−7^	2550	-0.001631 ± 0.0004120	7.73 × 10^−5^	-0.001594 ± 0.0002463	9.73 × 10^−11^
rs6586891	8	*LPL*	C>A	0.324	5291	-0.001554 ± 0.0003085	4.88 × 10^−7^	2559	-0.001583 ± 0.0004135	1.32 × 10^−4^	-0.001563 ± 0.0002474	2.62 × 10^−10^
rs8067076	17	*UNC13D*	T>G	0.48	5285	-0.001475 ± 0.0002909	4.11 × 10^−7^	2567	-0.0008020 ± 0.0003920	4.09 × 10^−2^	-0.001190 ± 0.0003326	3.47 × 10^−4^
rs6501843	17	*ACOX1*	A>G	0.297	5289	-0.001499 ± 0.0003146	1.94 × 10^−6^	2565	-0.0006614 ± 0.0004286	0.123	-0.001131 ± 0.0004159	6.55 × 10^−3^
rs2885819	1	*NR5A2*	G>T	0.279	5290	0.001513 ± 0.000321	2.50 × 10^−6^	2567	0.00001125 ± 0.0004336	0.979	0.0007906 ± 0.0007503	0.292
**LDLC:HDLC ratio**												
rs4420638	19	*APOC1*	T>C	0.114	5282	0.1624 ± 0.02959	4.26 × 10^−8^	2567	0.1356 ± 0.03853	4.39 × 10^−4^	0.1525 ± 0.02347	8.21 × 10^−11^
rs17445774	2	*C2orf47*	C>T	0.053	5278	0.1988 ± 0.04127	1.49 × 10^−6^	2567	0.1622 ± 0.05091	1.46 × 10^−3^	0.1843 ± 0.03206	9.01 × 10^−9^
rs2304072	5	*SLC25A48*	C>T	0.255	5291	0.09963 ± 0.02134	3.11 × 10^−6^	2566	0.04972 ± 0.02730	0.0687	0.07758 ± 0.02479	1.75 × 10^−3^
**non-HDLC:HDLC ratio**												
rs6589566	11	*APOA5*	A>G	0.215	5284	0.1601 ± 0.02758	6.78 × 10^−9^	2558	0.1091 ± 0.03744	3.60 × 10^−3^	0.1409 ± 0.02471	1.19 × 10^−8^

^a^Genes assigned closest to the SNP.

The beta (changes in lipid ratio per minor allele), se (standard error), and p-values were calculated by linear regression analysis in an additive model after adjusting for age, sex, and/or recruitment region: stage 1 with the KoGES population; stage 2 with the KDC population; combined analysis integrating association results from both the populations in a random effects model.

Abbreviations: Chr, chromosome; MAF, minor allele frequency; se, standard error; TG, triglyceride; HDLC, HDL cholesterol; LDLC, LDL cholesterol; APOA5, apolipoprotein A-V; LPL, lipoprotein lipase; UNC13D, unc-13 homolog D; ACOX1, acyl-CoA oxidase 1; NR5A2, nuclear receptor subfamily 5 group A member 2; APOC1, apolipoprotein C1; C2orf47, chromosome 2 open reading frame 46; SLC25A48, solute carrier family 25 member 48.

**Fig 1:**
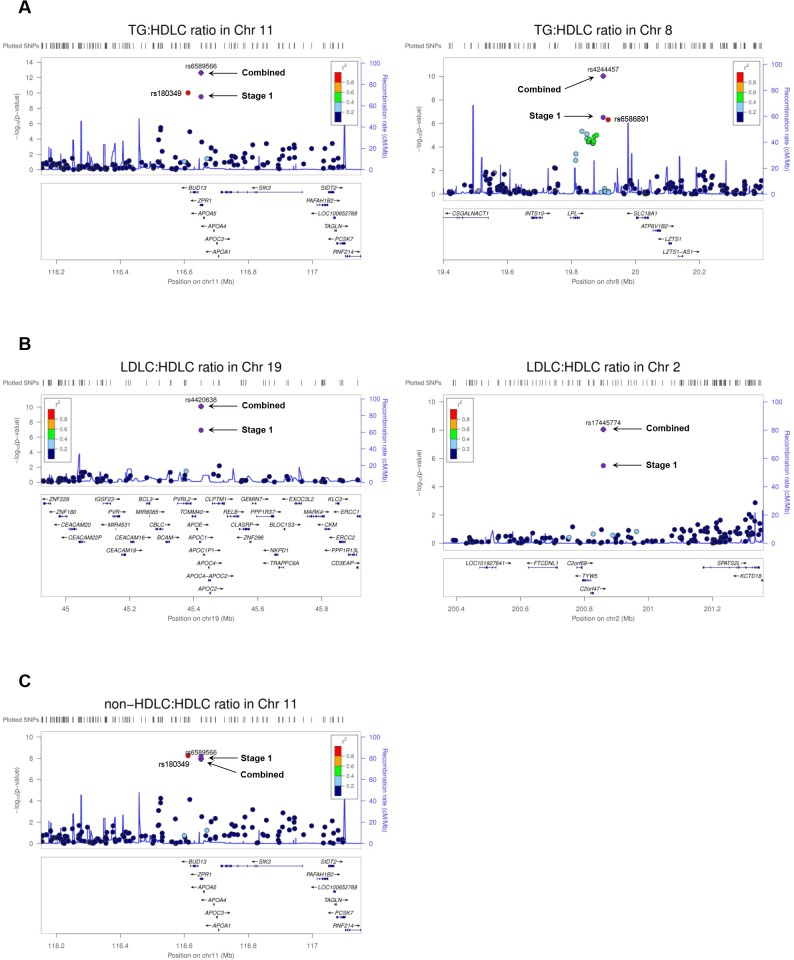
Regional plots of lipid ratios. The plot presents a 1-Mb region centering a peak SNP in each locus for lipid ratio: (A) for log[TG]:HDLC ratio, (B) for LDLC:HDLC ratio, and (C) for non-HDLC:HDLC ratio. Stage1 (blue circle): the association signal of a top SNP in stage 1; combined (blue diamond): the combined signal integrating association results from stage 1 (top SNP) and stage 2.

To confirm the associations of the variants with lipid ratios (except rs180349 due to deviation from HWE), we performed replication analysis (stage 2) in the KDC population. Of nine lipid ratio-associated variants, the associations of six variants in five loci (*APOA5*, *LPL* (lipoprotein lipase), unc-13 homolog D, *APOC1*, and *C2orf47* (chromosome 2 open reading frame 47)) were replicated (p < 0.05) (Table 2). After combining the minor allele effects both in stage 1 and in stage 2, three log[TG]:HDLC ratio-associated variants of *APOA5* and *LPL*, two LDLC:HDLC ratio-associated variants of *APOC1* and *C2orf47*, and one non-HDLC:HDLC ratio-associated variant of *APOA5* passed the genome-wide significance level, p < 5.0 × 10^−8^ (Table 2 and Fig 1). The minor allele of rs6589566 was found to be associated with increased log[TG]:HDLC ratio (β = 0.002078, p = 2.39 × 10‒^13^), while those of the *LPL* variants were associated with decreased log[TG]:HDLC ratio (rs4244457: β = −0.001594, p = 9.73 × 10‒^11^; rs6586891: β = −0.001563, p = 2.62 × 10‒^10^). The minor alleles of two variants, rs4420638 and rs17445774, were significantly associated with increased LDLC:HDLC ratio (rs4420638: β = 0.1525, p = 8.21 × 10‒^11^; rs17445774: β = 0.1843, p = 9.01 × 10‒^9^).

### Lipid ratio-associated variants according to constitutional types

A genetic discrepancy for cardiovascular risk exists between the TE (high risk) and NTE (low risk) types [17]. Therefore, we explored interactions between lipid ratio-associated variants and TE subgrouping, i.e., the TE and NTE types categorized based on the tertiles of the SCAT probability values for the TE constitutional type, by adding an interaction term to the linear regression model applied to all subjects. However, there were no significant interactions between the variants for three lipid ratios and TE subgrouping (P_TE-int_ > 0.05 in Table 3), as no remarkable differences in effect size between the two types, e.g., an opposite direction of the effect, were shown.

**Table 3:** Linear regression analysis for the lipid ratio in each constitutional subgroup

SNP	Chr	Gene^a^	Effect allele	Sub-group	Stage 1				Stage 2				Combined analysis	
					n	beta ± se	*P*-value	*P*_*TE-int*_	n	beta ± se	*P*-value	*P*_*TE-int*_	beta ± se	*P*-value
**log[TG]:HDLC ratio**														
rs6589566	11	*APOA5*	C	TE	1740	0.002242 ± 0.0006124	2.59 × 10^−4^	0.660	695	0.001303 ± 0.0009283	0.161	0.387	0.001956 ± 0.0005111	1.30 × 10^−4^
				**NTE**	**1741**	**0.001888 ± 0.0006137**	**2.13 × 10**^**−3**^		**694**	**0.002329 ± 0.0007673**	**2.50 × 10**^**−3**^		**0.002061 ± 0.0004792**	**1.71 × 10**^**−5**^
rs4244457	8	*LPL*	T	**TE**	**1739**	**-0.001605 ± 0.0005318**	**2.58 × 10**^**−3**^	0.495	**690**	**-0.001993 ± 0.0008201**	**1.54 × 10**^**−2**^	0.651	**-0.001719 ± 0.0004462**	**1.17 × 10**^**−4**^
				NTE	1737	-0.001100 ± 0.0005372	4.07 × 10^−2^		688	-0.002464 ± 0.0006509	1.67 × 10^−4^		-0.001730 ± 0.0006782	1.07 × 10^−2^
rs6586891	8	*LPL*	A	**TE**	**1743**	**-0.001591 ± 0.0005330**	**2.87 × 10**^**−3**^	0.393	**694**	**-0.001917 ± 0.0008288**	**2.10 × 10**^**−2**^	0.711	**-0.001687 ± 0.0004483**	**1.68 × 10**^**−4**^
				NTE	1742	-0.0009521 ± 0.0005410	0.0786		694	-0.002308 ± 0.0006481	3.95 × 10^−4^		-0.03270 ± 0.04049	0.419
**LDLC:HDLC ratio**														
rs4420638	19	*APOC1*	C	**TE**	**1738**	**0.1762 ± 0.05308**	**9.19 × 10**^**−4**^	0.440	**696**	**0.1789 ± 0.07495**	**1.73 × 10**^**−2**^	0.452	**0.1771 ± 0.04332**	**4.34 × 10**^**−5**^
				NTE	1738	0.1182 ± 0.05357	2.75 × 10^−2^		696	0.09651 ± 0.06473	0.136		0.1094 ± 0.04127	8.04 × 10^−3^
rs17445774	2	*C2orf47*	A	**TE**	**1741**	**0.1958 ± 0.06985**	**5.12 × 10**^**−3**^	0.895	**696**	**0.2267 ± 0.09964**	**2.32 × 10**^**−2**^	0.246	**0.2060 ± 0.05720**	**3.16 × 10**^**−4**^
				NTE	1734	0.1763 ± 0.07668	2.16 × 10^−2^		696	0.07145 ± 0.07993	0.372		0.1260 ± 0.05533	2.27 × 10^−2^
**non-HDLC:HDLC ratio**														
rs6589566	11	*APOA5*	C	**TE**	**1740**	**0.1272 ± 0.04776**	**7.83 × 10**^**−3**^	0.788	**695**	**0.1674 ± 0.07206**	**2.05 × 10**^**−2**^	0.284	**0.1395 ± 0.03981**	**4.60 × 10**^**−4**^
				**NTE**	**1742**	**0.1457 ± 0.04935**	**3.20 × 10**^**−3**^		**694**	**0.1509 ± 0.05829**	**9.82 × 10**^**−3**^		**0.1479 ± 0.03766**	**8.63 × 10**^**−5**^

^a^Genes assigned closest to the SNP.

The beta (changes in lipid ratio per minor allele), se (standard error), and p-values were calculated by linear regression analysis in an additive model, after adjusting age, sex, and/or recruitment region: stage 1 with the KoGES population; stage 2 with the KDC population; combined analysis integrating association results from both the populations in a random effect model.

Boldface letters indicate reproducible associations in all three populations.

P_TE-int_ value was assessed by performing an interaction analysis between SNP genotype and TE/non-TE type by adding an interaction term to the linear regression model.

Abbreviations: Chr, chromosome; se, standard error; TG, triglyceride; HDLC, HDL cholesterol; LDLC, LDL cholesterol; APOA5, apolipoprotein A-V; LPL, lipoprotein lipase; APOC1, apolipoprotein C1; C2orf47, chromosome 2 open reading frame 46; TE, Tae-Eum type; NTE, non-Tae-Eum type.

Because the TE type presented significantly higher lipid ratios in both KoGES and KDC populations when compared to the NTE type (Table 1), the associations of lipid ratio-associated variants were examined in constitutional subgroups. All five confirmed lipid ratio-associated variants in all the subjects presented significant constitution-consolidated association patterns (Table 3). That is, the minor allele effect of rs6589566 associated with increased log[TG]:HDLC was significant in the subgroup with the NTE type, whereas the effect of the SNP on non-HDLC:HDLC ratios remained significant in both TE and NTE types. The minor allele effects of the other four variants (rs4244457 and rs6586891 associated with decreased log[TG]:HDLC ratio and rs4420638 and rs17445774 associated with increased LDLC:HDLC ratio) remained significant in the subgroup with the TE type (Table 3).

## Discussion

Our GWAS was aimed at identifying the genetic factors associated with lipid ratios. We found a novel locus (*C2orf47-SPATS2L* (spermatogenesis associated serine rich 2 like) region) associated with the LDLC:HDLC ratio along with three known loci previously reported for individual lipid traits. In addition, we confirmed genetic discrepancy of lipid ratios according to the TE and NTE type.

In association tests between the TG:HDLC ratio and the SNPs, the strongest signal was observed for rs6589566 located downstream of *APOA5*, an SNP strongly correlated with 3′ UTR rs2266788 (calculated by Haploview version 4.2; r^2^ = 0.99 and D′ = 1.00 in Han Chinese in Beijing + Japanese population from HapMap 3 release #27) of *APOA5*. The minor allele of the 3’ UTR SNP reduces has-miR-3021 and has-miR-485-5p binding, resulting in reduced APOA5 expression and hypertriglyceridemia [28,29]. The second strong signal was detected for rs4244457 (highly correlated with rs6586891 showing the third strong signal; r^2^ = 0.97 and D’ = 0.94 in our study) located downstream of *LPL* that catalyzes the hydrolysis of lipoprotein TG and involves in the uptake of esterified lipids [30]. Further, rs4244457 was in strong LD (calculated by Haploview version 4.2; r^2^ = 0.48 and D′ = 0.90 in Han Chinese in Beijing + Japanese population from HapMap 3 release #27) with rs13702 in the 3′ UTR of *LPL*, which is associated with the change in blood TG and HDLC levels. The minor allele of rs13702 associated with decreased TG and increased HDLC disrupts the recognition site for has-miR-410 in the 3’ UTR of *LPL* and induces an increase in LPL expression [31].

The SNP rs4420638 close to *APOC1* has been found to be associated with higher LDLC and lower HDLC in previous reports [3,32]. rs4420638 also showed the strongest association with the LDLC:HDLC ratio in our study. However, the functional relationship between rs4420638 (or the correlated variants) and the change in the expression or activity of neighboring genes (*APOE*, *APOC1*, *APOC2*, and *APOC4*) remains unclear. The second strong signal for the LDLC:HDLC ratio was observed for rs17445774 close to *C2orf47*, which encodes uncharacterized protein, and is surrounded by formiminotransferase cyclodeaminase N-terminal like, *C2orf69*, tRNA-yW synthesizing protein 5, and *SPATS2L*. We searched lipid-SNP associations within the 1-Mb region around rs17445774 using two database tools, GRASP Search–v2.0.0.0 and PheGenI [33,34]. In total, 11 SNPs except rs17445774 were suggestively associated (1.75 × 10^−5^ < p < 9.90 × 10^−4^) with various lipid traits including TG, TC, LDLC, VDLC, HDLC, and ApoC3 levels in blood. Most of them had low LD with rs17445774, but two SNPs had strong LD (calculated by Haploview version 4.2; Both rs281787 and rs7565480 have r^2^ = 0.02 and D′ = 1.00 in Han Chinese in Beijing + Japanese population from HapMap 3 release #27) with rs17445774 and were found to be associated with ApoC3 levels (rs281787 p = 4.52 × 10^−5^ and rs7565480 p = 1.75 × 10^−5^) (S1 Table). ApoC3 is a component of remnant particles that inhibit the hydrolysis of TG-rich lipoproteins by LPL and the uptake of TG-rich lipoproteins by the liver, causing an increase in the TG level in the blood [35,36]. Moreover, C2orf47 is up-regulated 2.5-fold in human femoral atherosclerotic lesion, as determined in the gene expression analysis using microarrays [37]. These results indicate that this genomic region is genetically involved in the regulation of lipid metabolism.

The five SNPs of four loci satisfied the significance threshold (p-value < 5.0 × 10‒^8^) in this study, and three SNPs among them were also found to be associated with lipid levels in a previous report [17]. Upon comparing the results, we found that rs6589566 was more significantly associated with independent lipid levels (p < 2.00 × 10‒^16^ for increased TG levels; and p = 1.22 × 10‒^5^ for decreased HDLC levels, versus p = 2.39 × 10‒^13^ for increased TG:HDLC ratio). However, two SNPs were found to be more significantly associated with lipid ratios (rs4420638: p = 4.87 × 10‒^8^ for increased LDLC levels and p = 8.87 × 10‒^5^ for decreased HDLC levels, versus p = 8.21 × 10^−11^ for increased LDLC:HDLC ratio; rs6586891: p = 5.56 × 10‒^6^ for decreased TG levels and p = 9.39 × 10‒^9^ for increased HDLC levels, versus p = 2.62 × 10‒^10^ for decreased TG:HDLC ratio), although the present study had a smaller sample size than the previous one. This relatively higher significance suggests that the association test for lipid ratio is more effective in identifying genetic factors associated with lipid traits.

In the subgroup analysis according to constitutional type, several loci could be categorized into two groups according to their subgroup associations: (1) for the loci associated with both the TE and NTE types, the *APOA5* locus was associated with increased TG:HDLC ratio in the NTE type and increased non-HDLC:HDLC ratio in both the TE and NTE types. (2) For the loci associated only with the TE type, one locus (*LPL*) was associated with decreased TG:HDLC ratio, and two loci (*APOC1* and *C2orf47*) were associated with increased LDLC:HDLC ratio. Therefore, the TE type may be more susceptible to cardiometabolic risks caused by genetic elements compared to the NTE type, since the effects of most SNPs from the genome-wide scan were significant only in the TE type. This genetic discrepancy is consistent with the clinical discrepancy for cardiometabolic risks reported in a previous study [26].

One limitation of our study is that we did not analyze the associations between CHD risk and lipid-ratio SNPs including the *C2orf47* SNP, owing to lack of clinical information for CHD in the studied population. Therefore, we cannot conclude that the newly identified SNPs also play a significant role in CHD development.

In conclusion, we confirmed that the known loci associated with lipid levels were also associated with lipid ratios. Furthermore, a relationship between the *C2orf47* locus and the LDLC:HDLC ratio was newly discovered. Our study is significant in the discovery of this association of the *C2orf47* locus with the LDLC:HDLC ratio, given that the locus has a small effect on single-lipid phenotypes and has been overlooked in conventional single-lipid studies. With regard to the constitutional type, most SNPs exert genetic influences in the TE type. In the future, association studies for lipid ratios should be aimed at broadening the genetic perspective on cardiovascular diseases caused by atherogenic dyslipidemia.

## Supporting Information

S1 Fig Quantile–quantile plots for each lipid ratio. (A) For log[TG]:HDLC ratio, (B) LDLC:HDLC ratio, and (C) non-HDLC:HDLC ratio.
(TIFF)

S2 Fig Manhattan plots of genome-wide association analyses from stage 1. The ‒log_10_(*P*) values are plotted against chromosomal positions: (A) for log[TG]:HDLC ratio, (B) LDLC:HDLC ratio, and (C) non-HDLC:HDLC ratio. The red line indicates the cut-off p-value: 5.0 × 10^−6^.
(TIFF)

S1 Table Lipid-associated SNPs in the 1-Mb region around rs17445774. Using two search engines, lipid-associated SNPs were searched in the 1-Mb region around rs17445774. SNP positions were represented according to GRCh38.p2. r2 and D' values were calculated by Haploview version 4.2 using two reference genotype data: (1) Japanese from 1000 genome phase 3 data and (2) Han Chinese in Beijing + Japanese from HapMap release #27 data. Genotype data of rs10497847 could not be downloaded from HapMap and 1000 genome data.
(XLSX)
