# Supplementary figures and images for: Genome-Wide Association Study Reveals Four Loci for Lipid Ratios in the Korean Population and the Constitutional Subgroup
Source: PLoS One. 2017 Jan 3;12(1):e0168137. doi: 10.1371/journal.pone.0168137 (PMC5207643; doi:10.1371/journal.pone.0168137)

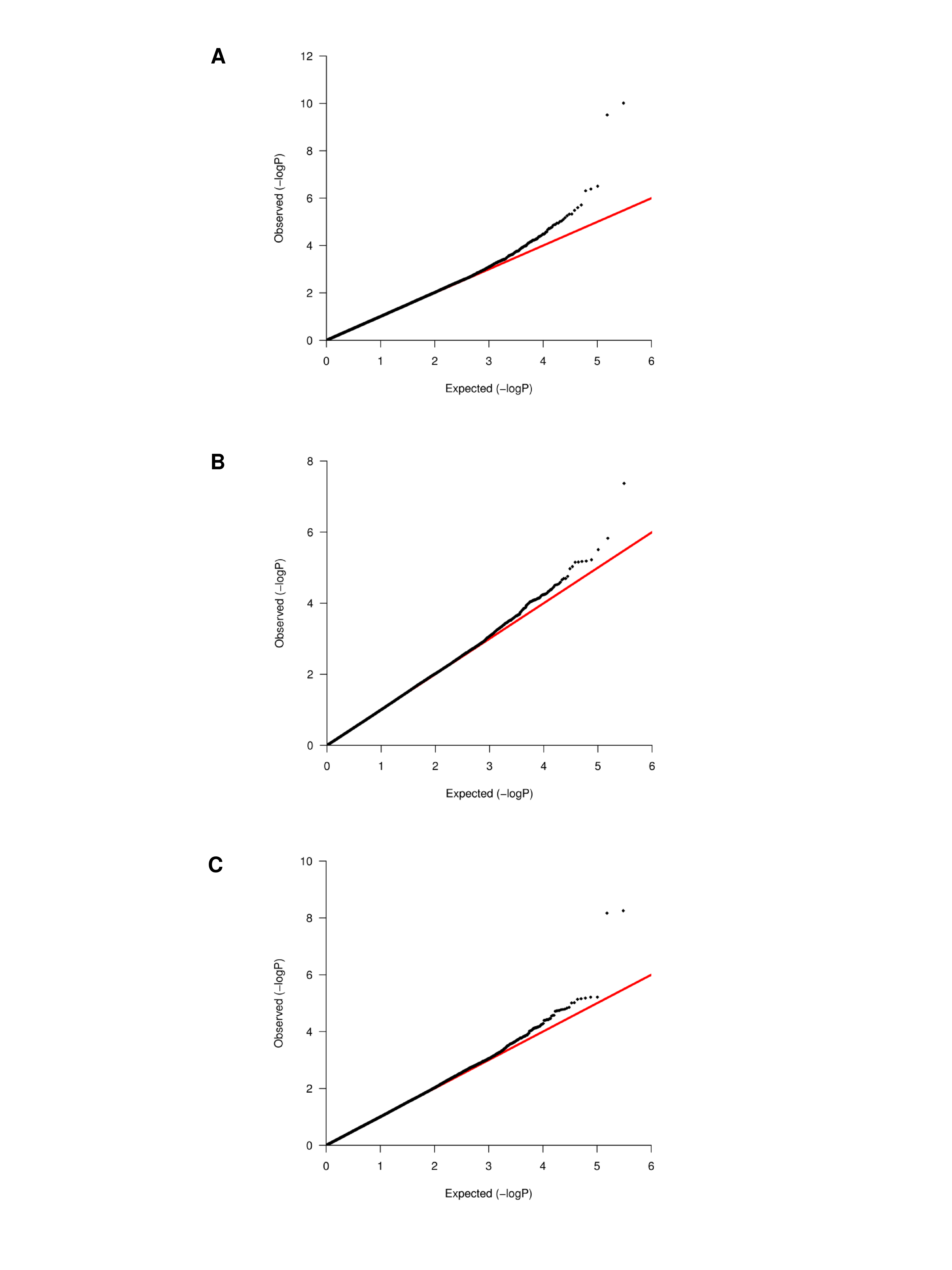

Supplement: S1 Fig — (A) For log[TG]:HDLC ratio, (B) LDLC:HDLC ratio, and (C) non-HDLC:HDLC ratio. (TIFF) [file pone.0168137.s001.tiff]

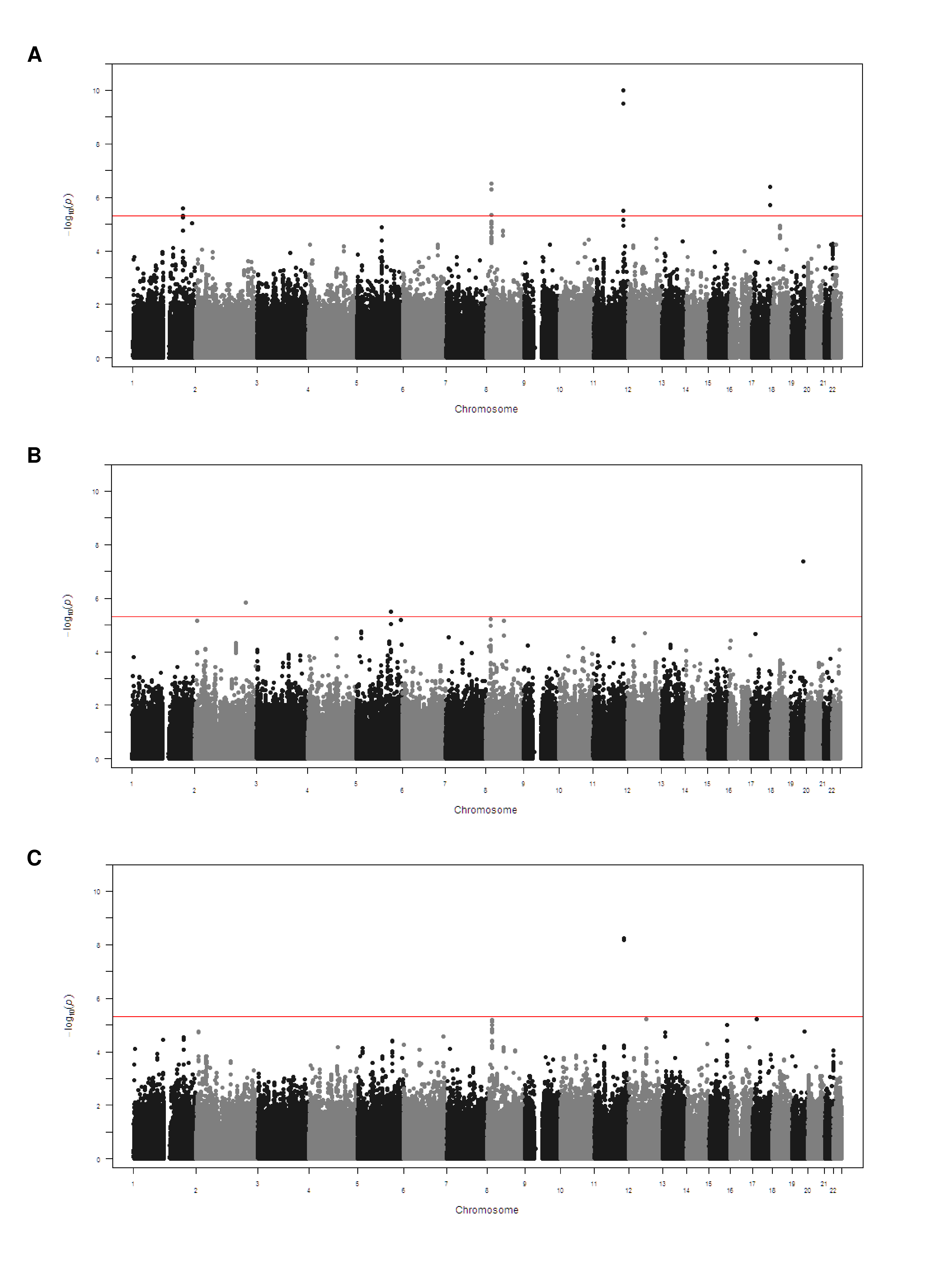

Supplement: S2 Fig — The ‒log10(P) values are plotted against chromosomal positions: (A) for log[TG]:HDLC ratio, (B) LDLC:HDLC ratio, and (C) non-HDLC:HDLC ratio. The red line indicates the cut-off p-value: 5.0 × 10^−6^. (TIFF) [file pone.0168137.s002.tiff]
